# Hepatocellular Carcinoma: An Uncommon Metastasis in the Orbit

**DOI:** 10.1155/2020/7526042

**Published:** 2020-02-26

**Authors:** Maria-Nikoletta Protopapa, Maria Lagadinou, Theodoros Papagiannis, Charalambos A. Gogos, Elena E. Solomou

**Affiliations:** University of Patras Medical School, Rion 26500, Greece

## Abstract

Hepatocellular carcinoma (HCC) represents the most common type of primary cancer of the liver and is associated with poor prognosis. It is the most common cause of death in cirrhotic patients and in different studies was shown as the third most common cause of cancer-related deaths worldwide. Each year, approximately half a million people are diagnosed with HCC. In recent decades, the prognosis of patients with HCC has improved because more cases are diagnosed and treated at early stages; high-risk patients (i.e., with chronic HBV or HCV infection) are followed more often for the possibility of HCC, and novel treatment options such as locoregional therapy are used with better overall results. The extrahepatic metastases represent a poor prognostic factor. The most common sites of metastasis in advanced hepatocellular carcinoma are the lung (44%), portal vein (35%), and portal lymph nodes (27%). Also, intra-abdominal lymph nodes and bones are common sites. Orbital metastases rarely occur, representing the 3-7% of orbital masses. These metastases are usually found in advanced tumor stages. The mechanism of metastasis to the orbit is difficult to determine. A hematogenous route, as for other primary neoplasms of the abdomen, may be suspected. Tumor cells may circulate through the vena cava, beyond the pulmonary filter to the heart, and finally be distributed to the orbital region through the arterial systemic circulation. We describe herein a case of an adult male with liver cirrhosis due to alcohol abuse who presented with concomitant diagnosis of HCC and orbit metastasis.

## 1. Introduction

Hepatocellular carcinoma (HCC) represents the most common primary cancer of the liver and the most common cause of death in cirrhotic patients. Each year, approximately half a million people worldwide are diagnosed with HCC [1, 2]. It is often observed in the 6th to 7th decade of life, and hepatitis B is the most frequent predisposition factor. With early diagnosis and effective available new treatment options (curative resection, ablation, and transplantation), the median survival time of patients is more than 5 years.

Prevalence of metastatic HCC is 18% with the lung being the most frequent site [3]. The extrahepatic metastases represent a poor prognostic factor. The most common sites of metastases in advanced hepatocellular carcinoma are the lung (44%), portal vein (35%), and portal lymph nodes (27%). Also, intra-abdominal lymph nodes and bones are common sites. Orbital metastasis rarely occurs [4].

We describe herein a case of an adult male with liver cirrhosis due to alcohol abuse who presented with concomitant diagnosis of HCC and orbit metastasis.

## 2. Patient Information

A 53-year-old man was first admitted to the ophthalmology department of our hospital because of the presence of a nodule on the left side of the forehead associated with visual loss on the same site. The patient reported no other associated symptoms. His personal history included chronic alcohol abuse and cirrhosis without any treatment or clinical monitoring. During his hospitalization, the patient underwent a computer tomography scan of the brain and was found with a retrobulbar mass, which had displaced the ipsilateral orbit and had infiltrated the orbit, frontal bone, and dura. The patient was referred to the internal medicine department for further investigation. This was the first time that we examined the patient since he had never sought medical advice for his condition.

Physical examination revealed icteric tinge of the skin and conjunctiva, left extraocular noncompact nodule in the forehead, ascites, signs of cirrhosis, and leg swelling. Culture of the ascitic fluid was negative, but the cytological examination revealed then the presence of HCC cells in the ascitic fluid. Blood work revealed high levels of alpha fetoprotein, increased bilirubin, and increased oxaloacetic and pyruvic transaminases.

Further imaging analysis with magnetic resonance of the brain showed a large lesion of the left orbital, which repelled the eyeball downward and inward and leached out the upper rectus, corroded the roof of the orbit, and filtered the dura. Extended alcoholic encephalopathy was also observed. A biopsy of the orbital mass was performed, which confirmed the HCC metastasis. The patient was only given radiation therapy since he refused any further treatment with chemotherapy, and he was discharged with little improvement.

## 3. Discussion

Orbital metastases are rare and represent only the 3-7% of orbital masses. This metastasis is usually found in advanced tumor stages and may precede the initial diagnosis of the primary site of the neoplasm [5]. The most common neoplasm associated with this kind of metastases is lung cancer, followed by prostate cancer in men and breast cancer in women [1, 6], but can also be seen in genitourinary and gastrointestinal tract cancers.

We reported the case of a rare metastasis of HCC in the orbit. Clinically diagnosed cases of HCC to the orbit are rare. A review of the relevant Chinese and English language medical literature by Chen et al. identified only 39 cases of HCC metastasis to the head reported since 2009. Of these 39 patients, 11 exhibited periorbital metastasis [2]. Of the 11 cases with HCC metastasis to the orbit published since then, all were male. Approximately 5.0% of the liver carcinomas showed infiltration also to the central nervous system [7].

HCC presenting with signs and symptoms related to distant metastases without abdominal discomfort or palpable liver masses is quite uncommon, representing less than 5% of the cases [8]. Moreover, the orbit found with a space-occupying lesion is a rare site of metastasis and usually found in 3.9% of all ocular tumors. However, one should be aware of ophthalmological symptoms and an initial symptom (face bone pain) and therefore of an early detection of this metastasis. Surgical removal of the extrahepatic metastatic lesions may be an option [6]. Systemic treatment with sorafenib also appears to be an alternative in these difficult-to-treat cases [9].

The mechanism of metastasis to the orbit is difficult to determine. A hematogenous route, as for other primary neoplasms of the abdomen, may be suspected. Tumor cells may circulate through the vena cava, beyond the pulmonary filter to the heart, and finally be distributed to the orbital region through the arterial systemic circulation. Other authors have reported that tumor cells can reach the head and neck by bypassing the lungs, possibly through the vertebral venous plexus of Batson [6]. The examination of the metastasis mechanism is of great importance in these cases particularly when the nearby bones are also involved. In our patient, extensive bone involvement in direct proximity to the eye was also observed.

Accurate staging of hepatocellular carcinoma (HCC) is critical for guiding optimal treatment options, especially nowadays with the extensive usage of sorafenib. However, there is limited data about when and how to screen extrahepatic metastases for newly diagnosed HCC patients, especially for patients without signs and symptoms [10]. Although orbital metastases are rare, one should be aware of this clinical form and rare metastasis site, which is associated with poor prognosis. All efforts are directed in palliating the patient's symptoms [6].

## Supplementary Materials

Supplementary Materials Figure 1 shows photographs of orbital metastasis showing the presence of a nodule on the left side of the forehead associated with visual loss on the same site.
