# Supplementary material for: Hepatocellular Carcinoma: An Uncommon Metastasis in the Orbit
Source: Case Rep Oncol Med. 2020 Feb 26;2020:7526042. doi: 10.1155/2020/7526042 (PMC7061109; doi:10.1155/2020/7526042)
Supplement: Supplementary Materials — Figure 1 shows photographs of orbital metastasis showing the presence of a nodule on the left side of the forehead associated with visual loss on the same site. [file 7526042.f1.docx]

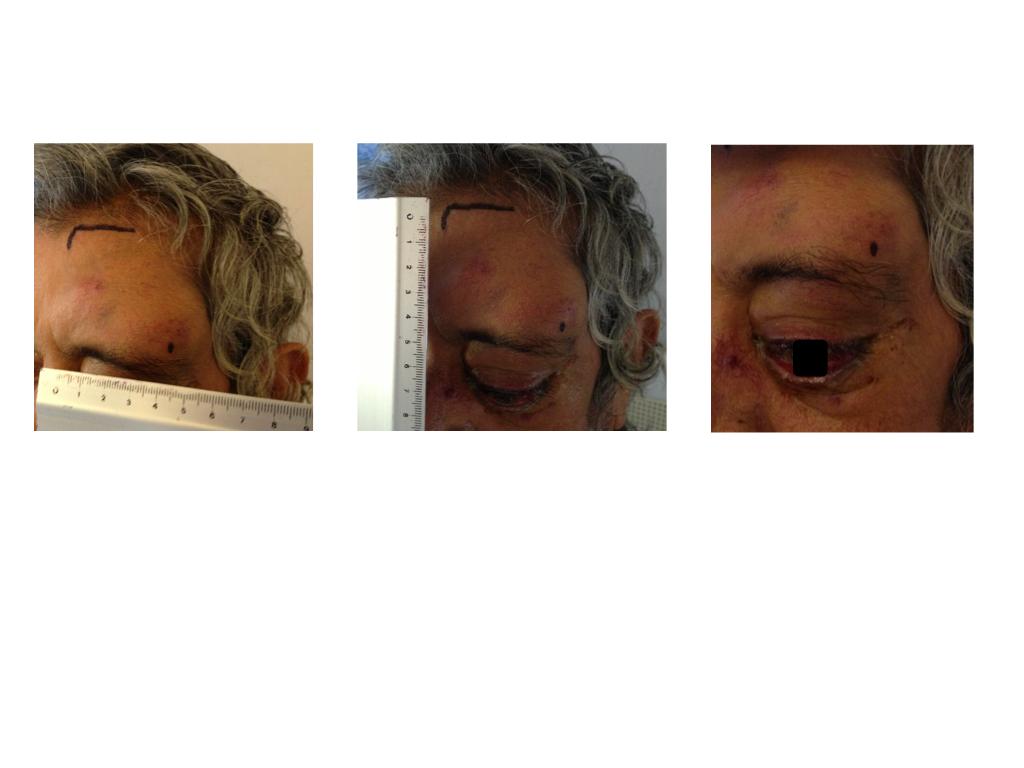


**Figure 1:** here are photographs showing the orbital metastasis as the presence of a nodule on the left side of the forehead associated with visual loss on the same site
